# Mechanisms of lung and diaphragmatic protection by high PEEP in obese COVID-19 ARDS: role of the body mass index

**DOI:** 10.1186/s13054-022-04051-8

**Published:** 2022-06-17

**Authors:** Joaquin Pérez, Javier Hernan Dorado, Emiliano Navarro, Caio C. A. Morais, Matías Accoce

**Affiliations:** 1 Intensive Care Unit, Sanatorio Anchorena San Martín, Provincia de Buenos Aires, Perdriel 4189, Villa Lynch, San Martín, Argentina; 2 Intensive Care Unit, Hospital de Agudos Carlos G. Durand, Ciudad Autónoma de Buenos Aires, Argentina; 3 Centro del Parque, Ciudad Autónoma de Buenos Aires, Argentina; 4 Divisão de PneumologiaInstituto Do Coração (Incor), Hospital das ClínicasFaculdade de Medicina, Universidade de São Paulo, São Paulo, Brazil; 5 Departamento de Fisioterapia, Universidade Federal de Pernambuco, Recife, Brazil; 6 Intensive Care Unit, Hospital de Quemados “Arturo Umberto Illia”, Ciudad Autónoma de Buenos Aires, Argentina; 7 Universidad Abierta Interamericana. Facultad de Medicina Y Ciencias de La Salud, Buenos Aires, Argentina

## To the Editor:

Obesity increases the risk of requiring mechanical ventilation (MV) and developing acute respiratory distress syndrome (ARDS) in patients with coronavirus 2019 (COVID-19) [1].

Spontaneous breathing (SB) may potentially compromise lung and diaphragmatic protection in COVID-19-induced ARDS [2]. In recruitable models, high positive end-expiratory pressure (PEEP) was shown to render SB less injurious [3]. Nonetheless, the effects of high PEEP have been scarcely studied in clinical scenarios. Previous literature suggests that obese subjects might benefit from higher PEEP and these positive effects might be proportional to the obesity grade [4, 5].

We assessed whether the beneficial effects of high PEEP on lung and diaphragmatic protection depend on body mass index (BMI) in intubated obese patients recovering from COVID-19-associated ARDS.

This is an exploratory analysis of a physiological study conducted at Sanatorio Anchorena San Martín, Argentina. Consecutive adults with COVID-19-associated ARDS, BMI > 30 kg/m^2^, intubation > 48 hs and SB in pressure support for > 2 hs were included.

The pressure support level was titrated by the attending physician. Esophageal pressure (P_es_) was measured with an air-filled (1 mL) esophageal balloon (MBMed®-BA-A-008, non-latex) according to manufacturer recommendations after balloon’s correct positioning. Airway pressure, flow, volume and P_es_ were recorded by a dedicated system (FluxMed, MBMed®).

Our primary endpoints were the changes in muscular pressure (P_mus_), pressure–time product per-minute (PTP_min_) and dynamic driving transpulmonary pressure (ΔPL_dyn_) from low-to-high PEEP and its correlation with BMI. Data were collected 10 min after changing PEEP (5–15cmH_2_O). Respiratory variables averages were computed based on respiratory cycles of the last 30–60 s of each PEEP step considered stable. In 16/21 patients, chest wall elastance (E_cw_) was assessed before SB was present. In the remaining, E_cw_ was measured applying a hyperventilation maneuver by increasing the pressure support until observing positive P_es_ deflections. All traces were analyzed using Biopac Student Lab PRO®.

Correlations were performed with Spearman's rank test. To assess the differential effects of high PEEP, we analyzed subgroups based on a commonly used BMI cutoff (≥ 35 kg/m^2^) [4]. A linear mixed-effects regression model for repeated measures was fitted to assess interaction between PEEP (5–15cmH_2_O) and BMI (≥ or < 35 kg/m^2^). *P* values were adjusted by post hoc Tukey’s correction. A two-tailed *P* ≤ 0.05 was considered statistically significant. Data were analyzed with R4.0.3 software.

We studied 21 patients (age, median [IQR, 25th–75th]: 56 years [IQR, 51–67]; BMI: 34.3 kg/m^2^ [IQR, 31.6–40.4]). At admission, 85% had moderate ARDS and 3 had mild ARDS. All the patients required neuromuscular blockers and 62% received prone ventilation.

At inclusion, the patients had 5 MV days [IQR, 3–7] and remained ventilated for additional 11 days [IQR, 5–20]. The PaO_2_/FiO_2_ was 214 mmHg [IQR, 183–252], pressure support was 8cmH_2_O [IQR, 5–10], and inspired oxygen was 0.40 [IQR, 0.35–0.50]; the Richmond Agitation–Sedation Scale was − 3 to − 4.

**Fig. 1:**
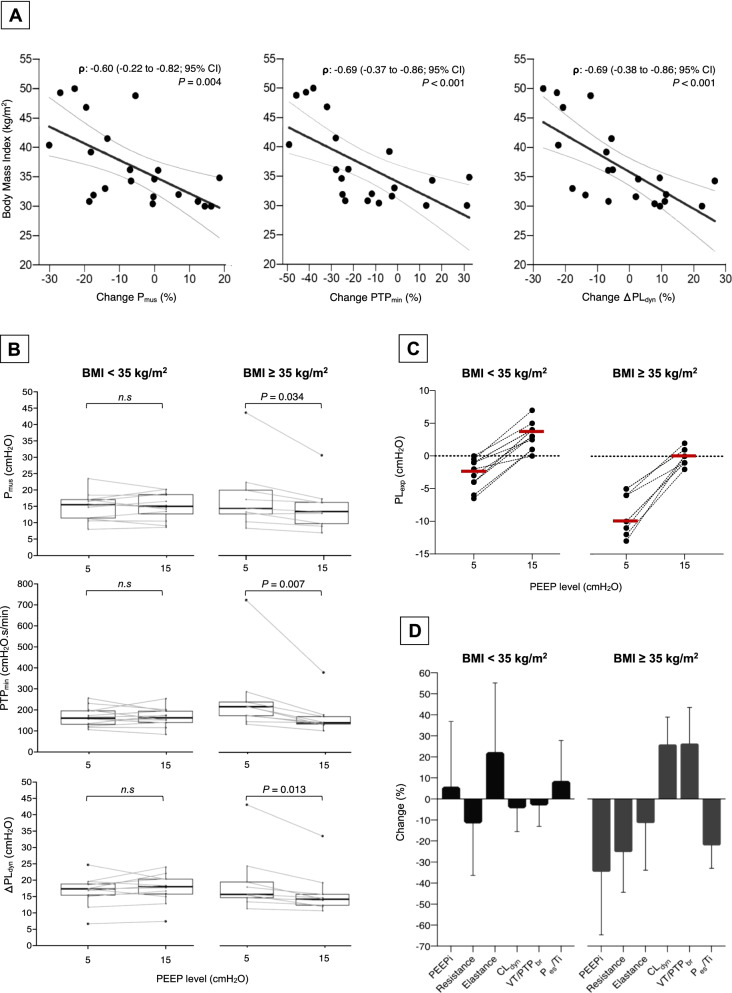
**A** Correlation between BMI and changes (%) in P_mus_, PTP_min_, and ΔPL_dyn_; **B** P_musc_ (upper), PTP_min_ (middle) and ΔPL_dyn_ (lower) with PEEP 5 and 15cmH_2_O; **C** end-expiratory transpulmonary pressure at PEEP 5 and 15cmH_2_O; **D** mean (95% CI) percentage of change in PTP-associated components (intrinsic PEEP, resistance, elastance), dynamic lung compliance (CL_dyn_), neuromuscular ventilatory coupling expressed as tidal volume-to-pressure–time product per breath (VT/PTP_breath_) and intensity of respiratory drive expressed as esophageal pressure to neural inspiratory time (P_es_/Ti). In panels **B**, **C** and **D**, the patients are divided in subgroups according to BMI < 35 kg/m^2^ (n = 12; median BMI 31.8 [IQR, 30.7–33.3] kg/m^2^) and ≥ 35 kg/m^2^ (n = 9, median BMI 43.6 [IQR, 38.5–48.9] kg/m^2^)

The BMI significantly correlated with changes in P_mus_, PTP_min_ and ΔPL_dyn_ (Fig. 1-A) We found an interaction between BMI and PEEP regarding to changes in P_mus_ (F_(1,19)=_6.2;*P* = 0.022), PTP_min_ (F_(1,19)=_7.4, *P* = 0.013) and ΔPL_dyn_ (F_(1,19)=_10.4, *P* = 0.004). Only the subgroup with BMI ≥ 35 reduced the P_mus_ (mean difference (95%CI): − 3.3cmH_2_O (− 1.5 to − 6.1)), PTP_min_ (− 94.5 cmH_2_O.s/min(− 23.3 to − 165.7)) and ΔPL_dyn_ (− 3.2cmH_2_O(− 1.6 to − 5.7)) with high PEEP (Fig. 1-B). The results remained significant after excluding an outlier from the severely obese subgroup. In these patients (BMI ≥ 35), the end-expiratory transpulmonary pressure (PL_exp_) was more negative at low PEEP and became closer to 0cmH_2_O at high PEEP (Fig. 1-C). The optimization of lung–diaphragmatic protection was explained by reduction of PTP-associated components, improvement in dynamic lung compliance (CL_dyn_), neuromuscular ventilatory coupling and respiratory drive (Fig. 1-D).

We demonstrate that the obesity grade greatly influences the beneficial effects of PEEP on relevant monitoring variables directly linked to the lung and diaphragmatic protection.

In obese subjects, the increased pleural pressure may reduce end-expiratory lung volume and become PL_exp_ negative, causing atelectasis and airway closure [4, 5]. Alveolar and airways collapse may alter ventilation distribution and cause occult pendelluft, resulting in local/global overdistension [3]. This might impose higher loads on the respiratory muscles, jeopardizing the SB even more [5].

Our findings indicate that these pathological mechanisms may play a role during SB in obese COVID-19 ARDS patients and can be counteracted by PEEP [4, 5]. Nonetheless, despite fulfilling obesity criteria (BMI > 30 kg/m^2^), not all patients benefited from high PEEP. Accordingly, the least obese subgroup did not experience work of breathing unloading and deteriorated lung mechanics, neuroventilatory coupling and respiratory drive, suggesting that lung overdistension was probably induced. In this context, we suggest that P_es_ should be used to titrate PEEP during assisted ventilation to avoid the potential harms associated with unnecessarily high PEEP levels, taking a slightly positive PL_exp_ as a reasonable target [4].

Our study is mainly limited by the small sample size and the lack of direct measurements of lung–diaphragmatic injury. Additionally, PEEP steps were not random and the time spent during each PEEP level was short.

In summary, considering the complexity of SB in COVID-19-induced ARDS [2], high PEEP might be considered as a potentially useful tool to provide lung and diaphragmatic protection particularly in selected individuals with BMI ≥ 35 kg/m^2^, where the obesity-associated mechanical alterations might be specially exacerbated.

## Data Availability

The datasets used and analyzed during the current study are available from the corresponding author on reasonable request.

## Abbreviations

MV: Mechanical ventilation
ARDS: Acute respiratory distress syndrome
COVID-19: Coronavirus disease 2019
SB: Spontaneous breathing
PEEP: Positive end-expiratory pressure
BMI: Body mass index
P_es_: Esophageal pressure
P_mus_: Muscular pressure
PTP_min_: Pressure–time product per minute
ΔPL_dyn_: Peak dynamic driving transpulmonary pressure
PaO_2_/FiO_2_: Arterial oxygen pressure to inspired oxygen
P_es_/Ti: Esophageal pressure to neural inspiratory time
VT/PTP_breath_: Tidal volume-to-pressure–time product per breath
CL_dyn_: Dynamic lung compliance
n.s: No statistically significant
IQR: Interquartile range
